# Evaluation of the Effectiveness and Efficiency of the East African Community Joint Assessment Procedure by Member Countries: The Way Forward

**DOI:** 10.3389/fphar.2022.891506

**Published:** 2022-07-05

**Authors:** Nancy Ngum, Jane Mashingia, Margareth Ndomondo-Sigonda, Stuart Walker, Sam Salek

**Affiliations:** ^1^ Department of Clinical and Pharmaceutical Sciences, School of Life and Medical Sciences, University of Hertfordshire, Hatfield, United Kingdom; ^2^ African Union Development Agency, New Partnership for Africa’s Development (AUDA-NEPAD), Johannesburg, South Africa; ^3^ East African Community Secretariat, Arusha, Tanzania; ^4^ Centre for Innovation in Regulatory Science, London, United Kingdom; ^5^ Institute of Medicines Development, Cardiff, United Kingdom

**Keywords:** EAC joint assessment procedure, East African Community Medicines Regulatory Harmonization, benefits, challenges, effectiveness, efficiency, joint regulatory assessment

## Abstract

**Background:** For almost a decade, the East African Community has implemented the Medicines Regulatory Harmonization (EAC-MRH) programme among its member states to harmonise technical requirements and standards for medical products regulation, jointly conduct scientific review of medical product dossiers to assess safety, efficacy and quality, inspect pharmaceutical manufacturing sites and streamline decision-making processes. This initiative enables the cost-effective use of limited resources and efficient and effective delivery of regulatory services to be determined, thus instilling transparency and accountability in all stakeholders, optimising the pharmaceutical market and economic development and improving access to safe, high-quality, effective medicines in the region. The aim of this study was to evaluate the effectiveness and efficiency of the current operating model of the EAC-MRH initiative, including challenges faced and to identify opportunities for improvement.

**Methods:** The Process Effectiveness and Efficiency Rating (PEER) questionnaire, which was used to identify the benefits, challenges, and suggestions for improving performance of EAC-MRH initiative, was completed by assessors representing seven EAC authorities in the joint assessment procedure. Semi-structured interviews were also carried out to validate the responses.

**Results:** This initiative has been of considerable value as it moves toward achieving its main objectives of shorter timelines for approval of medicines, information sharing among regulators and capacity building for assessments, resulting in quicker access and increased availability of medicines for patients in the region. However, the key challenges identified that have hindered effectiveness and efficiency were the lack of a centralised submission and tracking system; inadequate human resources, manufacturers’ failure to submit the exact same dossier to all countries of interest; lack of an integrated information management system; lack of information on national medical regulatory authority or EAC websites; and challenges in monitoring and tracking assessment reports.

**Conclusion:** The use of a robust information technology system for the central tracking of EAC products is essential to address the identified challenges and improve regulatory effectiveness and efficiency. One central point for payment is needed to expedite the process and to ensure transparency and the availability of information on decision making on national and regional websites. Other key strategies for enhancement include improving the capacity of assessors, work and information sharing and a coordination mechanism for the regional joint assessment, with the eventual establishment of a regional medicine agency.

## 1 Introduction

The East African Community (EAC) is a regional inter-governmental organization of seven national medicines regulatory authorities (NRAs) consisting of six partner states, namely the Republic of Burundi, Republic of Kenya, Republic of Uganda, Republic of Rwanda, Republic of South Sudan and the United Republic of Tanzania. The United Republic of Tanzania is composed of the Tanzania Mainland and Tanzania Zanzibar. According to the EAC-MRH Secretariat 2021 report, all seven agencies have been benchmarked by WHO. One out of the seven NRAs is still working towards attaining Maturity Level 1, Four NRAs are at Maturity Level (ML) 1 and one NRA has attained ML3. All the seven agencies are at different levels of implementation of their Institutional Development Plans to improve their maturity levels. No NRA in the region currently has PIC/S membership, although the NDA of Uganda is preparing to apply for membership. No NRA has observer status in the ICH. Furthermore, TMDA, NDA, PPB, and Rwanda FDA have provided assessors for the WHO PQ medicines assessments (Copenhagen sessions). In addition, inspectors from NDA Uganda have worked under the WHO PQ Rotational Fellowship for Inspections.

Countries in this region have experienced the circulation of substandard and falsified medicines (Ndomondo-Sigonda et al., 2020). Currently, the prevalence of these products in Africa is estimated at 25%–30% and represents a major threat to public health, negatively impacting the growth of the African pharmaceutical sector and its overall contribution to economic development and resulting in numerous deaths (Ndomondo-Sigonda et al., 2020). According to Roth and colleagues, about 10% of medicines in low- and middle-income countries are substandard and falsified and the lack of timely access to good quality and effective medicines has been a major challenge in Africa (Roth et al., 2018).

The review and registration of medical products is one of the key functions of regulatory authorities that influences access to medical products (Sithole et al., 2021a). There are several bottlenecks that impact the registration of medical products in African countries by pharmaceutical companies (Narsai et al., 2012). One of these is the lack of capacity, in which 30% of NRAs do not have the necessary expertise to conduct key regulatory functions (Keyter et al., 2020a). Hence, there is a need to strengthen medicines regulatory systems in this continent.

Given the capacity differences in regulating medical products in African Member States, it is important to note that the African Union (AU) Member States and Regional Economic Communities (RECs) are making significant efforts to improve access to safe, quality, and efficacious medical products through strengthening and harmonising medicines regulatory systems. Studies show that the reluctance from companies manufacturing medical products to register their products in African markets is one of the major factors delaying access to medicines (Sillo et al., 2020). Reasons for this reluctance is due to the lengthy application process, the time, expense, and effort needed for this registration process in each NRA (Sillo et al., 2020). To improve access to safe, quality and effective medical products, the EAC joint assessment project was established in 2012, to assist in facilitating the market authorisation application process for manufacturers through a faster review of applications in the region.

A key strategy proposed by Roth and colleagues is to leverage convergence and reliance efforts (Roth et al., 2018). According to the Centre for Innovation in Regulatory Science, many NRAs are now using reliance as a mechanism to minimise duplication, maximise limited resources, build capacity and improve timely access to safe, high-quality, effective medical products (CIRS, 2021). In their study on the impact of reliance on the review process of the South African Health Products Regulatory Authority (SAHPRA), Keyter and associates showed that the introduction of reliance pathways; that is, the use of the abridged review model by the SAHPRA, led to 68% faster timelines for the approval of medicines and improved patient access to medical products (Keyter et al., 2021).

Six authorities studied by Sithole and colleagues are using reliance (verification and abridged reviews) and this will hopefully improve access to medical products in these countries (Sithole et al., 2021a). Another comparative study of the registration process of the medicines control authority of Zimbabwe (MCAZ) with Australia, Canada, Singapore, and Switzerland indicated that reliance is key in agencies that rely mainly on industry fees for sustainability like MCAZ (Sithole et al., 2021b). These authorities are already demanding a high fee for applications for products to enter the market and do not have the opportunity to increase these fees again to support resources for regulatory reviews. On the other hand, agencies with funds from government can increase resources to improve performance. Reliance is therefore a useful mechanism to assist agencies in these instances to improve regulatory performance as they will focus their limited resources on medical products that have not been reviewed elsewhere.

However, regulatory authorities and manufacturers might not have sufficient experience in using reliance to register new medicines as it is still a relatively new concept (CIRS, 2021). Barriers and enablers in implementing reliance models identified in a study of pharmaceutical company perceptions indicated that the main strengths were shorter approval timelines and reduced requirements. In the same study, identified weaknesses of reliance included the lack of unredacted assessment reports, long submission lag times and pathways that were not fully adopted (CIRS, 2021). In addition to these challenges for reliance, a study on reliance in South Africa, identified a lack of benefit-risk assessments; the perception that reliance would lead to loss of expertise, especially in less resourced agencies; and inadequate transparency in decision-making processes as key hurdles (Keyter et al., 2020b).

**FIGURE 1:**
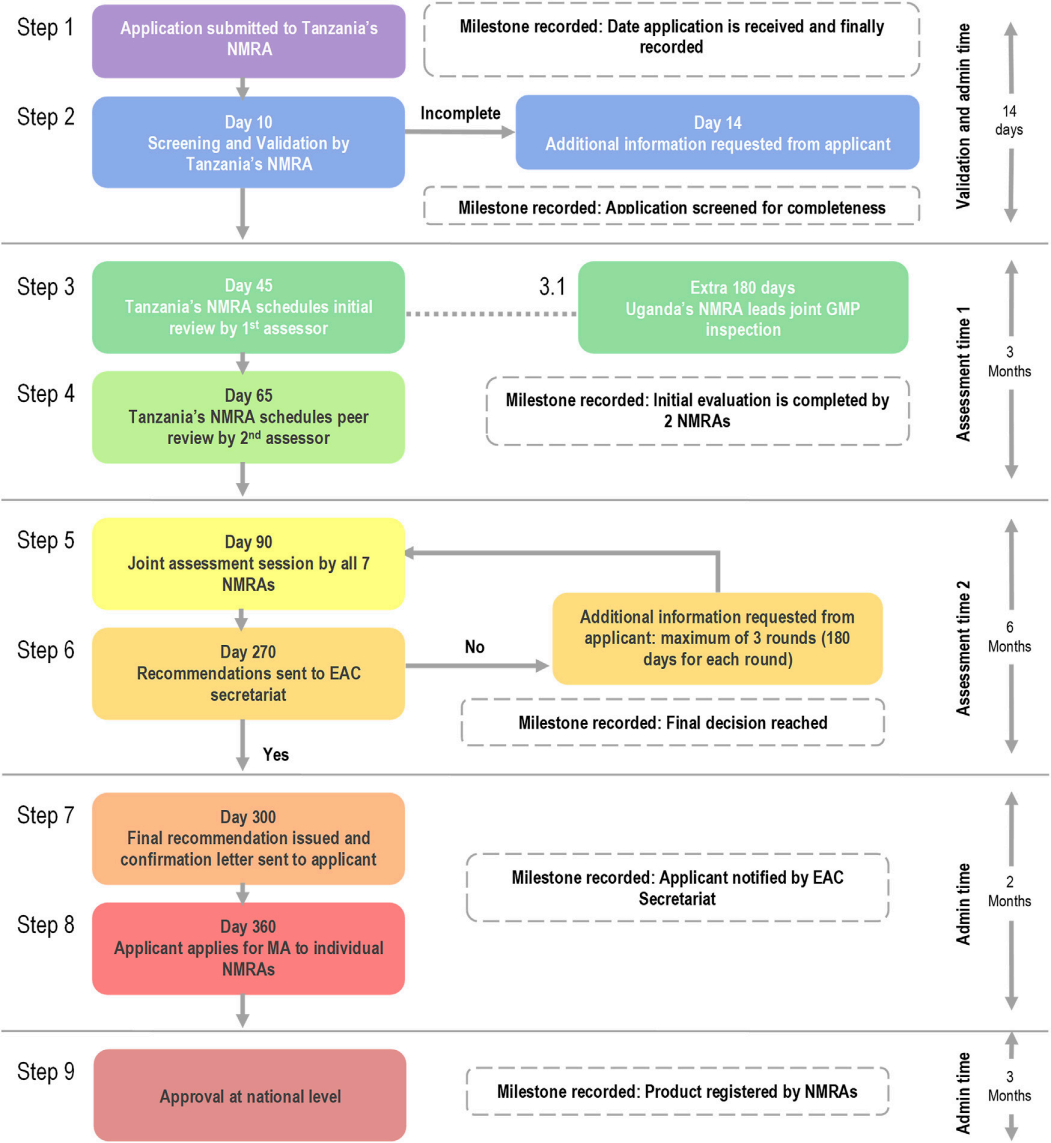
Review process map and milestones for EAC joint assessment procedure.

The EAC joint medicines regulatory process consists of a joint assessment of dossiers of medical products and a joint inspection of manufacturing sites. This process started in 2015 and can be described using 9 steps (Figure 1).

Step 1 starts with the submission of the application to the lead NRA, the Tanzania Medicines and Medical Devices Authority (TMDA). In Step 2, the lead authority screens the application to check for completeness, including the good manufacturing practice (GMP) Status (Day 10). For Step 3, TMDA schedules the initial review, which also includes the GMP inspection led by the Uganda National Drug Authority (NDA; Day 45) and the GMP inspection could take another 180 days. In step 4 (day 65), an initial review is completed by two NRAs and by day 90, a joint assessment session is held (Step 5) with all representatives from the seven NRAs. At this stage a list of questions or queries are sent to the applicant when appropriate for applicant response. A maximum of three rounds is implemented, with each expected to last about 180 days. In step 6, documents are compiled and recommendations from the joint assessment are sent to the EAC Secretariat (Day 270). By day 300 (step 7), the final recommendation is issued, and a confirmation letter sent to the applicant. In step 8 (day 360), the applicant is expected to apply for marketing authorisation to individual NRAs, with approvals at national levels (step 9) and which should take place within 90 working days. Unlike the approach of the European Medicines Agency (2016) where it is mandatory for countries to register medicines approved through the centralised processed, in Africa, this is not mandatory.

With the launch of the EAC-MRH programme, the EAC authorities have made substantial progress in reducing timelines for registration of medical products using the joint review process. A study of the EAC-MRH pilot phase (2012–2017) by Mashingia and colleagues found that registration timelines were reduced from 24 months to 8–12 months for products reviewed using this process (Mashingia et al., 2020).

There has been a drive within regulatory authorities in recent years to re-engineer their processes for improved effectiveness and efficiency and this often begins with a baseline evaluation of the current process to identify strengths and weaknesses. *Effectiveness* can be defined as “doing the right thing”, often measured by the value derived by customers or stakeholders of an organisation’s processes or services, while *efficiency* can be defined as “doing the right things right”, which saves an organisation time and resources. The aim of this study was to evaluate the effectiveness and efficiency of the current operating model of the EAC-MRH initiative, including the challenges it faces as well as identifying opportunities for improvement.

## 2 Study Objectives

1) Obtain the views of the individual medicines regulatory authorities of the EAC-MRH initiative about the performance of the joint assessment initiative to date
2) Identify the challenges experienced by individual authorities throughout the life cycle of the EAC-MRH initiative
3) Determine the strengths and weaknesses of the initiative
4) Identify the ways of improving the performance of the joint assessment initiative
5) Envisage a strategy for moving forward to improve effectiveness and efficiency

## 3 Methods

### 3.1 Study Participants

The PEER questionnaire was completed by seven NRAs of the EAC joint assessment: Pharmacy and Poisons Board (PPB), Republic of Kenya; National Drug Authority Uganda (NDA), Republic of Uganda; Rwanda Food and Drugs Authority (Rwanda FDA), Republic of Rwanda; Burundi Food and Medicines Regulatory Authority (ABREMA), Republic of Burundi; Drug and Food Control Authority (DFCA), Republic of South Sudan; Tanzania Medicines and Medical Devices Authority (TMDA) and Zanzibar Medicines and Medical Devices Authority (ZMDA) of the United Republic of Tanzania.

### 3.2 Questionnaire Development and Validation

A Process Effectiveness and Efficiency Rating (PEER) questionnaire was developed by the authors to identify the views of regulators on the benefits, challenges and opportunities for improving performance of EAC-MRH initiative. The PEER questionnaire (Supplementary Material S1) was validated by carrying out a pilot study with two authorities to establish its practicality, applicability and content validity.

Semi-structured interviews using a checklist (Supplementary Material S2) were carried out with each authority to validate their responses to the questionnaire. The main respondents were the seven assessors representing their agencies in the EAC-MRH joint assessments. The Heads of the seven agencies validated the responses by the assessors. The interview provided flexibility and a further opportunity for the respondents, as they were able to give more open-ended answers to some questions. Some sections in the questionnaire were clarified, challenges in completing the questionnaire were discussed and the benefits of the study were acknowledged. To ensure confidentiality, the questionnaire was marked as “confidential” and participants were also informed about this during the interviews. Consent was obtained from the participants on the information that was to be shared and to minimise bias, participants reviewed the final study report. Responses and explanations were made in some sections of the questionnaire. To ensure accuracy in capturing the entire interview sessions, they were audio recorded.

## 4 Results

For ease of understanding, the results are presented in five parts:

1) Authority resources.

2) Benefits of the EAC-MRH Initiative.

3) Challenges of the EAC-MRH Initiative.

4) Improving Performance of the work-sharing programme.

5) Strategies for moving forward.

### 4.1 Part 1: Authority Resources

This part of the questionnaire provided insight into the human resources availability and size of the participating NRAs.

The total number of staff for each of the seven responding agencies ranged from 33 to 338; the number of reviewers for marketing authorisation applications ranged from 4 to 50; while the number of reviewers that participate in the EAC joint assessments from these authorities ranged from 4 to 20. (Table 1).

**TABLE 1:** National Medicines Regulatory Authority information on human resources.

Measure	ABREMA Burundi	PPB Kenya	Rwanda FDA Rwanda	DFCA South Sudan	TMDA Tanzania	NDA Uganda	Zfdaa Zanzibar
Total number of staff in your agency	33	187	196	16	338 plus 48 temporary staff	287	150
Number of reviewers of marketing authorisation applications	8	15	15	4	50	30	10
Reviewers participating in the EAC joint assessments	4	6	4	4	19	20	5

Only two agencies kept a separate record of applications received for assessment under EAC-MRH while five authorities did not. Reasons given for not having such a record included inadequate capacity as well as manufacturers not filing applications in all authorities for the EAC procedure. One authority reported that although they did not have a separate record, they could use their system to filter EAC applications, as segregation of applications is possible for new applications, but the old ones must be retrieved manually as such data is not appropriately archived.

### 4.2 Part 2: Benefits of the EAC-MRH Initiative

This part focused on the benefits and strengths of the joint process for recommending the registration of products to NRAs, manufacturers, and patients.

**FIGURE 2:**
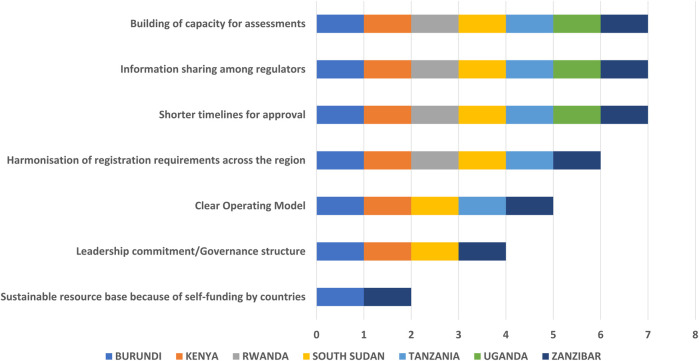
Benefits of the EAC-MRH initiative.

Shorter timelines for approval, information sharing among regulators, and building capacity for assessments were highlighted by all seven authorities as the main benefits of the EAC initiative (Figure 2). Building capacity for assessments was indicated by all as a considerable benefit, which was especially apparent in less-resourced agencies. Some agencies alluded to the fact that they never had assessors before the EAC-MRH but now have been able to rectify their situation because of the EAC joint assessment process. Harmonisation of registration requirements across the region was another benefit selected by six NRAs. Leadership commitment had improved significantly because of the collaboration with EAC, World Health Organization (WHO) and NRAs.

All NRAs indicated that they have a pool of expert reviewers and this and the priority review of EAC products were the strengths of the EAC process at a country level. Regular committee meetings enabling the timely registration of products after EAC recommendation was another strength (5/7) while four NRAs indicated resource savings were a benefit.

**FIGURE 3:**
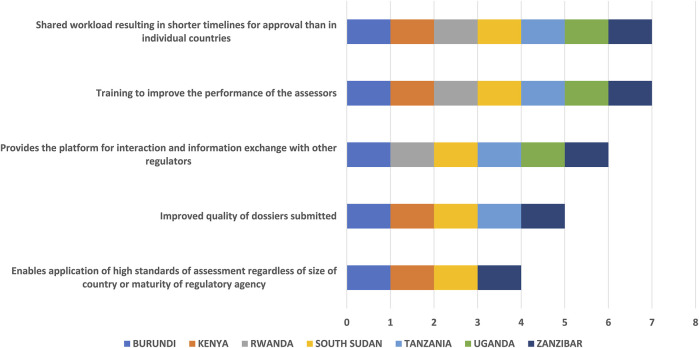
Benefits of the EAC-MRH initiative to regulators.

This initiative has benefitted regulators in training, improved the performance of assessors and facilitated shared workloads, resulting in shorter timelines for approval than in individual countries. It has also provided a platform for interaction and information exchange with other regulators. However, this interaction occurs only during assessment sessions and there is no post-assessment exchange (Figure 3).

There is a reduced burden for applicants, who compile only one dossier (modules 2–5) for submission to multiple countries and receive the same list of questions from multiple NRAs, enabling the compilation of a single response package, leading to savings in time and resources. Shorter timelines for approval compared with that of individual countries has enabled access to various markets at the same time.

The EAC-MRH procedure has allowed quicker access to quality-assured medicines and increased the availability of medicines for patients in the region. However, this initiative has not reduced the prices of medicines, as some generic products still maintain high prices. Furthermore, because applicants do not always apply to all agencies participating in the EAC-MRH joint assessment, the benefits of the EAC initiative for patients will only apply to some NRAs in the region.

### 4.3 Part 3: Challenges of the EAC-MRH Initiative

The major challenge to the initiative identified by the authorities is the lack of a centralised submission and tracking system. Also, as mentioned, manufacturers may only apply to NRAs in their countries of interest.

The lack of detailed information on the process for applicants was expressed by four respondents, with the concern that applicants sometimes apply to both the EAC and to the NRA.

One NRA respondent indicated unequal workloads among the NRAs as a challenge, as dossiers are allocated to the well-resourced NRAs while less-resourced NRAs are given query responses from applicants to process. These assignments are necessary because new applications and complex dossiers cannot be assessed by the less resourced NRAs, but they result in an increased workload for authorities with greater resources compared with those that are less resourced.

**FIGURE 4:**
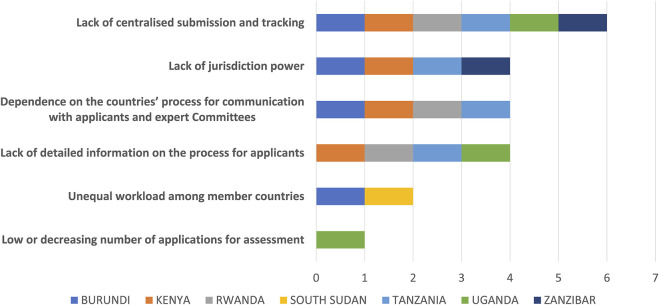
Challenges of the EAC-MRH initiative.

Lack of sharing of consolidated (aggregated) information by the lead country, particularly for consolidated assessment reports was also cited as a major challenge. Assessors often struggle to get reports after the assessment sessions are completed, because, although there is an assumption that countries safely retain reports after assessment, this is not the case (Figure 4). Following an interview, one of the respondents stated that: “Only the list of products approved are shared without the report. This delays the process of registration in order to get the report as it is needed for national registration”.

Most NRAs mentioned inadequate human resources as the key challenge at a country level and even one of the well-resourced NRAs expressed the need for more assessors to adequately handle the number of applications received for assessment.

Failure by manufacturers to follow the requirement to submit the exact same dossier to all countries of interest is also a major challenge for authorities. Poor record keeping and tracking of EAC-MRH products at national level is another hurdle for some agencies, as they do not maintain a separate record of applications received for assessment under EAC-MRH programme, and applicants sometimes submit applications for joint review to the EAC and then submit the same application at a national level. This creates duplicative communication, with parallel assessments conducted at both country and regional levels.

The unpredictability of applications causes scheduling inefficiencies, sometimes warranting the convention of unscheduled meetings to cover unanticipated applications or the postponement of scheduled meetings if enough applications have not been received.

Although the EAC-MRH work can provide learning experience to assessors, it is not recognised as part of regulatory authority work to be carried out during working hours, which was seen by authorities as an issue.

**FIGURE 5:**
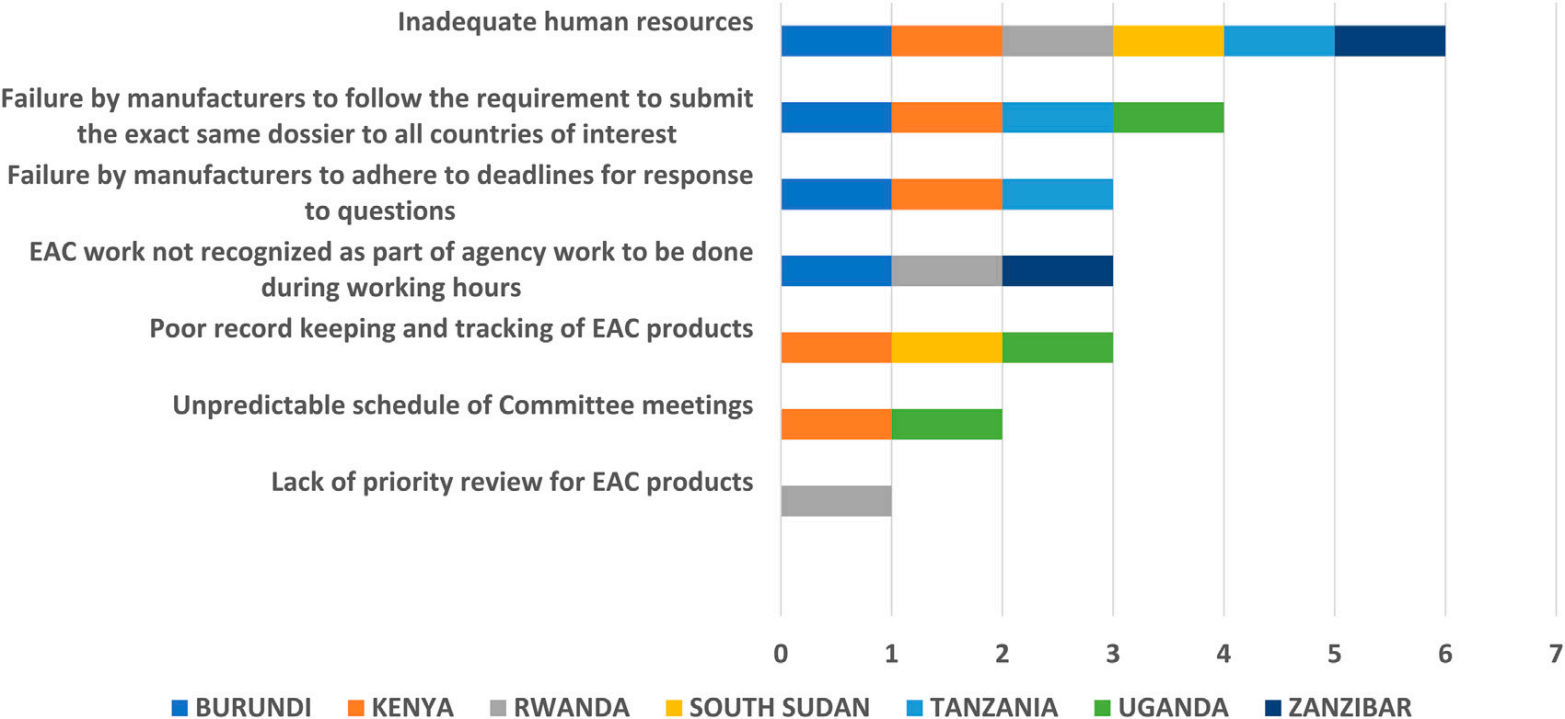
Challenges assessing EAC-MRH products at country level.

Failure by manufacturers to adhere to deadlines in response to questions is a challenge and due to this delay, some NRAs may provide marketing authorisation without the nomination of the local technical representative by the manufacturer as required (Figure 5). Because the EAC conducts a stringent assessment, applicants may apply to less stringent countries (NRAs) to get their products registered. However, applicants do not have full information on the application process, as there is no guidance on how to submit applications on the EAC website and there is lack of clarity about the process for submission and follow up in each NRA. Applications should go to the lead NRA for EAC assessments, but some applicants still send applications to other NRAs. There are significant differences in time to the implementation of EAC-MRH recommendations by the NRA which could be caused by the lack of a centralised system for payment of the application fees to all EAC NRAs. Finally, differing labelling requirements in participating countries was also highlighted as one of the challenges faced by applicants.

### 4.4 Part 4: Improving the Performance (Effectiveness and Efficiency) of the Work-Sharing Programme

**FIGURE 6:**
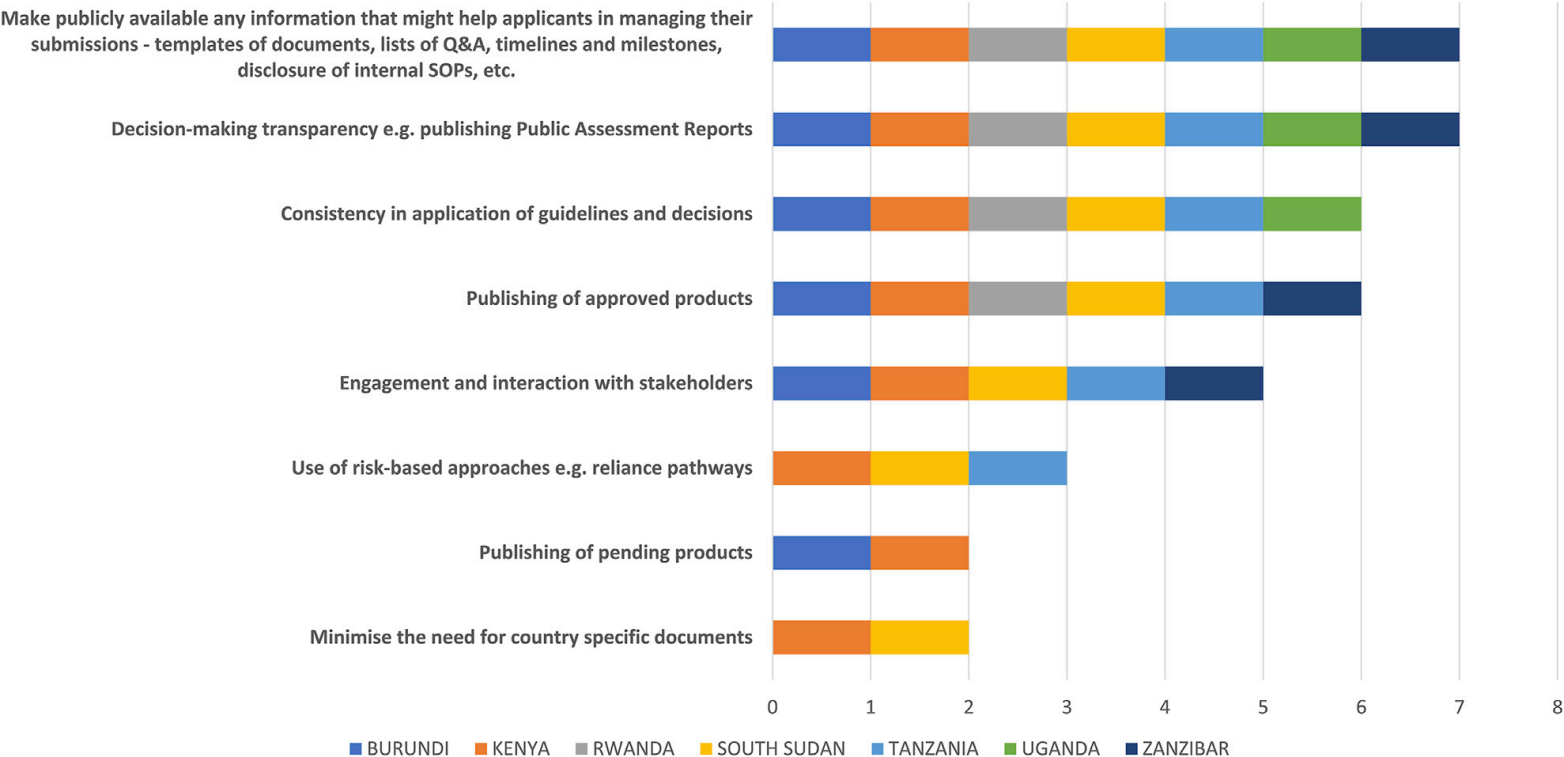
Ways to improve effectiveness of the EAC-MRH initiative.

Determining the views of the regulators in improving effectiveness and efficiency of the EAC-MRH initiative was an important part of this study. The top three ways to improve effectiveness identified by respondents were 1) decision-making transparency; for example, publishing public assessment reports or making any information publicly available that might help applicants in managing their submissions such as templates, lists of questions and answers, timelines and milestones; 2) disclosure of internal SOPs; and 3) consistency in application of guidelines and decisions (Figure 6).

Other suggestions for improvement included ensuring good record keeping for application and report traceability and sharing access to the consolidated assessment reports and query responses with NRAs by the host country NRA.

The host country for GMP should also share inspection reports with the EAC secretariat, sharing product approval letters with the focal persons. This information should be uploaded to the portals in order to facilitate compliance with NRA requirement for proof of how products are approved through the EAC procedure. This information is typically provided to the applicants, but a copy should also be requested to be sent to the NRA to assist scheduling of the final committee meetings at the national level.

**FIGURE 7:**
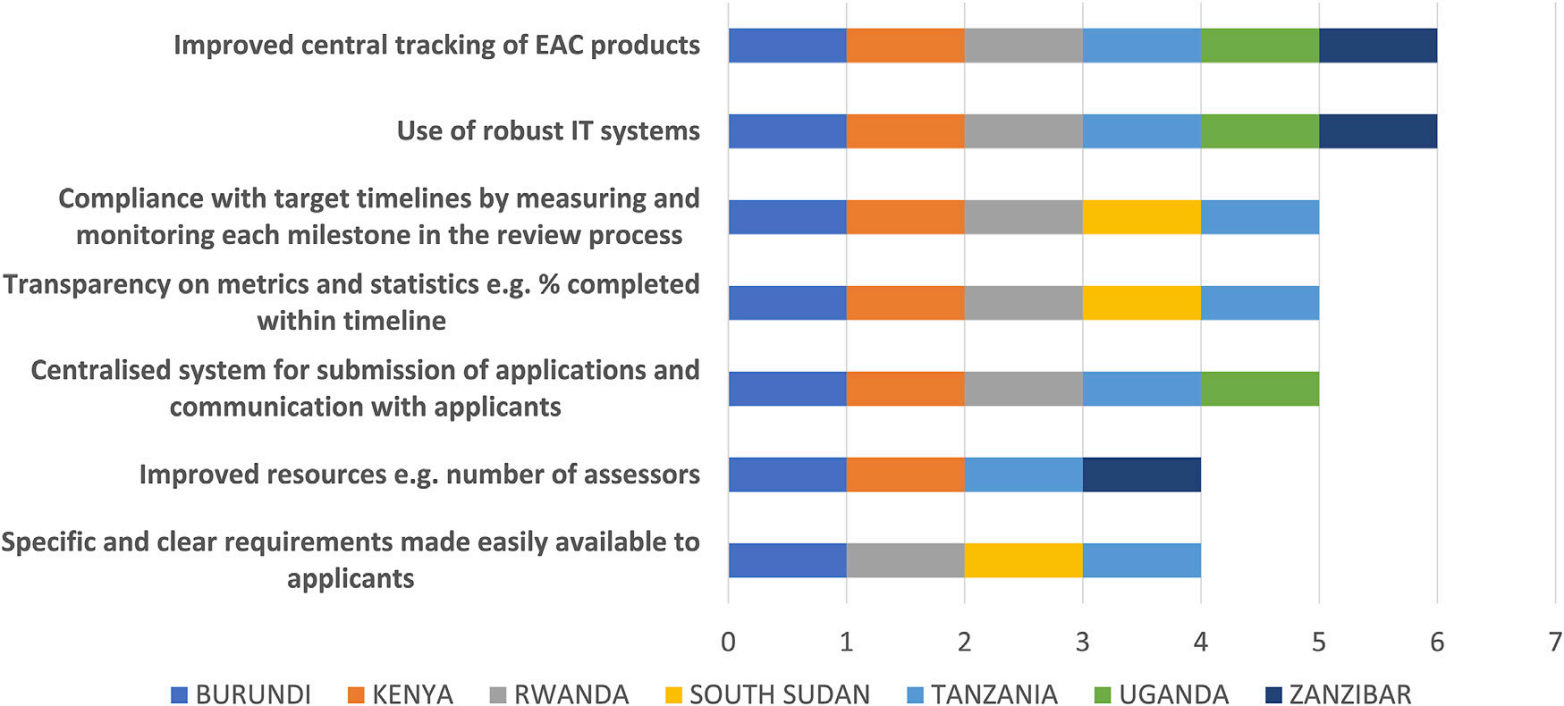
Ways to improve efficiency of the EAC-MRH initiative.

The top five ways identified to improve the efficiency of the EAC initiative were (Figure 7) 1) improved central tracking of EAC products; 2) the use of robust IT systems; 3) compliance with target timelines by measuring and monitoring each milestone in the review process; 4) transparency on metrics and statistics and 5) a centralised system for submission of applications and communication with applicants.

### 4.5 Part 5: Strategies for Moving Forward

The following proposals were suggested to improve the EAC operating model. First, continue with the current operating model and establish an EAC integrated information management system to manage and process applications; second, continue with the current operating model but provide full information on the process, including timelines and milestones as well as approved products on every participating country’s website and on the EAC website. The third option, to continue with the current operating model unchanged was not considered appropriate.

Other strategies proposed that would strengthen the initiative going forward were.

#### 4.5.1 Capacity Building

The EAC should support and work closely with less-resourced regulatory authorities to build their capacity to the level of better-resourced NRAs in the region. Following an interview, one of the respondents stated that: “A major request here is for the EAC to facilitate the process of weak NRAs in order to improve from the basic to the intermediate level and so they eventually become experts”. The NRAs should be supervised after the joint review processes to make sure they are doing the right thing. Although the expectation is that the EAC experts are well versed with regulatory subject matters after training, this is not always the case, and supervision may still be needed. In addition, training is currently needed for new assessors as many trained experts have left their agencies. Finally, the EAC joint assessment should be included among the workload of the authority to avoid delays in the assessment process.

#### 4.5.2 Improving Work and Information Sharing

Improved communication within the EAC NRA is critical and this can be achieved by sharing the final assessment reports of the approved products with all NRAs. Because authorities must access the reports for the national registration process, sharing only the list of approved products without the reports results in unnecessary delays. The development of a robust IT system for the EAC-MRH that can be used for tracking and uploading dossier as well as a repository for reports is required. Apart from Tanzania NRA, the agencies in the region do not have an appropriate IT infrastructure, although Kenya is in the process of developing such a system.

The availability of financial or technical support will assist the development of an efficient information management system.

#### 4.5.3 EAC–MRH Coordinating Mechanism

The authorities agreed that the EAC-MRH coordinating mechanism at the secretariat level should be strengthened. Legal procedures should be developed to enable the EAC secretariat to perform some functions such as the collection of fees and charges for joint activities that are not currently performed by NRA such as active pharmaceutical ingredient master file certification procedures and inspection of clinical research organisations. Regularly sharing research findings, providing regulatory training, and the exchange of experts for mentorship, coaching and capacity building of EAC NRAs would be helpful. The need for all seven NRAs in the region to be operating with similar standards is an important objective for developing competency. Experience has shown that manufacturers take applications to agencies with lower standards, as they will request fewer requirements and make the process easier than the EAC process. Therefore, it is important that NRAs in the region have the same standard as the EAC-MRH process. All NRAs in the region should encourage more companies to embrace the EAC-MRH initiative.

#### 4.5.4 Establishing a Regional Authority

Establishing a regional authority was reported to be the best strategy for improved performance, as it would promote innovation and access to new technologies; ensure all EAC NRAs have access to high-quality, safe and effective medicines; improve the quality of medicines and reduce sub-standard and falsified products in the region as well as improve regulatory expertise across the EAC; provide a global overview of the different regulatory developments at national and international levels as well as facilitating information sharing and best practices among regulatory experts.

The reasons for not establishing a regional authority cited by respondents included a need to strengthen the regulatory systems for all the EAC NRAs. As many of the authorities depend on the fees collected from the applicants to fund their operations, distributing the fees among the members states if the regional authority was established would present a challenge. It was further suggested that the region is not sufficiently mature yet for a regional agency; however, by establishing the EAC regional medicines authority, capacity building and existing collaboration among countries might be maximised. It was also stated that the establishment of EAC regional medicines authority is not necessary as the African Medicine Agency (AMA) will soon be coming into force; however, the mandate for the AMA depends on the support of the regional agencies. It is understood that the AMA will be regulating only complex molecules while NRAs and Regional Agencies will continue with evaluation of other essential medical products. Therefore, the AMA is not replacing the NRAs, but will complement and support their work.

## 5 Discussion

The aim of this study was to evaluate the effectiveness and efficiency of the current operating model of the East African Community Medicines Regulatory Harmonisation initiative including the challenges it faces as well as identifying opportunities for improvement.

The NRA acknowledged that the initiative has been of considerable benefit as it has moved toward achieving its main objectives, which are shorter timelines for approval of medicines, the existence of information sharing among regulators and building capacity for the agencies. The timely registration of products after EAC recommendation has been enabled by regular EAC committee meetings, shared workloads and the creation of a pool of expert reviewers, which has led to resource savings. Also, allowing applicants to compile one dossier for submission to multiple countries has enabled the industry to have simultaneous access to several markets. The strengths of this initiative have resulted in quicker access and increased availability of quality-assured medicines for patients in the region.

The median time for joint assessment in 2019 was reported to have decreased to 240 working days, demonstrating that the EAC joint assessment process was becoming more efficient (Mashingia et al., 2020). In the same study, registration timelines at the national level were reduced from 24 months to 8–14 months during the 2012–2017 time period (Mashingia et al., 2020). Giaquinto and colleagues also confirmed that one of the strengths of this initiative was the implementation of the joint assessment and work-sharing procedure with the introduction of the submission of one dossier by applicants to all EAC authorities (Giaquinto et al., 2020). The twinning programme was also identified as one of the key strengths of this initiative (Giaquinto et al., 2020).

However, several key challenges were identified that have affected the full realisation of the benefits of this initiative. They include the lack of a centralised submission and tracking system, with most agencies not having separate records of applications received for assessment under EAC-MRH, inadequate human resources, failure by manufacturers to follow the requirement to submit the exact same dossier to all countries of interest, lack of information on country or EAC websites, poor record keeping and tracking of EAC products, assessors not having access to reports after the joint assessment sessions, and the EAC-MRH work not recognised as part of the respective national authority workload.

The outcome of this study also has confirmed the findings from other authors. In a pilot study of the EAC-MRH, Mashingia and associates identified numerous challenges faced by the EAC harmonisation initiative. These included the difficulty for applicants tracking the progress of their applications as the system is not transparent in terms of timelines; inadequate follow-up to questions by both applicant and NRAs; delays in some products being granted marketing authorisation at the national level after the regional approval has been made; financial sustainability as well as submission of applications and fees by manufacturers to all EAC NRAs after the joint review process (Mashingia et al., 2020). Different capacities of NRAs affects trust, as sometimes authorities tend not to rely on the decisions of the new authorities in the region. Whilst harmonisation has had some benefits, it has impacted the less mature agencies who have not specialised, as they tend to rely on the mature agencies instead of building their own expertise. Other barriers highlighted in the study were lack of a legally binding framework amongst the NRA in the EAC; understaffing and staff turnover and less involvement by the heads of agencies in shaping the agenda of the harmonisation programme (Mashingia et al., 2020).

To address some of the weaknesses and improve effectiveness and efficiency, it is suggested that the use of a robust IT system to improve the central tracking of EAC products is essential. Ensuring the availability of information on decision-making transparency on the websites (national and regional) and establishing one central point for payment would also make the process faster. The lesson to be learned from the European Medicines Agency is that registration of medicines approved through the central process should be mandatory. With only one NRA in the region that operates at maturity level 3, improving the capacity of assessors as well as work and information sharing and the coordination mechanism for the regional joint assessment programme with the eventual establishment of the regional medicine’s authority would be key strategies for moving forward. The African Medicines Agency treaty came into force on 5th November 2021 after the 15th ratification instrument was deposited at the African Union Commission. Two EAC member states have ratified the AMA treaty. One of the mechanisms being put in place to operationalise AMA is the building of regulatory work force. The African Medicines Regulatory Harmonisation Initiative has been leading the work force development through the establishment of Regional Centres of Regulatory Excellence (RCOREs) and the medicines regulatory harmonisation programmes (Ncube et al., 2021). Giaquinto and colleagues are also of the view that transparency, responding to feedback from industry, meeting registration timelines, reliance and utilising metrics would further improve access to essential medical products in the region (Giaquinto et al., 2020). Charging its own fees as the initiative increases its scope and making joint regulatory decisions mandatory would assist in sustaining the initiative (Giaquinto et al., 2020). In their study on the evaluation of the review models and approval timelines of countries participating in the Southern African Development Community Medicines Regulatory Harmonization (SADC-MRH) project, Sithole and associates recommended that national regulatory systems be strengthened to equip them to fully participate in reliance initiatives such as Zazibona (Sithole et al., 2021a). This recommendation would also apply to the EAC-MRH joint assessment procedure, as countries in this region work towards relying on the reviews and decisions made by other agencies in order to fast track access to safe, high-quality and effective medicines by patients. The opportunity to implement a reliance strategy by regulatory authorities would improve transparency and accountability and take advantage of regulatory decisions through the utilisation of assessment reports. According to Keyter and colleagues, published assessment reports should include information on how the regulatory authority has analysed the benefits and risks of the medical product and made their final decision. The study recommends the use of a standardised approach to public assessment reports to improve communication on benefit-risk assessment, which in turn would support any reliance initiatives (Keyter et al., 2020a).

Arik and colleagues also proposed several approaches in the EAC Road Map 2020–2022 to address the challenges encountered in implementing the EAC-MRH project. These included having Regional Technical Officers, who are fully dedicated to regional activities, unlike the usual practice, in which NRA staff have had to take part on an ad hoc basis, with insufficient time allocated for regional activities, the establishment of a cooperation agreement, the introduction of a coordination fee to support regional assessments and inspections, as well as the expansion into new product areas (biologics, biosimilars) should be considered. A major proposal in the road map was the establishment of single autonomous authority for the region (Arik et al., 2020).

The key recommendations in this study to improve effectiveness and efficiency of the EAC-MRA joint assessment include:

1) Initiation of an industry cross-sectional study—A similar study should be conducted with the industry to obtain their perception of the joint assessment procedure so that there is a balanced view from both regulators and the industry.
2) Initiation of a longitudinal study**–**this would enable collection of efficiency and effectiveness data in order to demonstrate change (i.e., improvement) over time.
3) Measuring and monitoring timelines—The development of an integrated system for tracking applications for the regional initiative to monitor registration timelines of the products. NRAs should take full responsibility for tracking applications and recommended products for the EAC joint procedure. Also, An internal portal for information sharing by the assessors should also be made available to enhance post-assessment session interactions by regulators. This portal should also be used as a repository for reports. In addition, target timelines should be established for all the milestones including review time and applicant response time.
4) Availability of submission guidelines—The existing EAC-MRH programme and NRA websites should be enhanced with clear guidelines on the process of submission for the EAC procedure and follow up by each authority to improve the application process, transparency, accountability, and communication.
5) Training and capacity building**–**Continuous training of assessors should be conducted, as it would lead to staff retention and improvement in motivation, especially as there is high staff turnover within the authorities. The twinning programme should be reinstated, as it was of great benefit to the less resourced agencies.
6) The EAC-MRH coordination process–This should be strengthened to improve programme implementation and achieve the expected results. Sensitisation and awareness campaigns should be conducted to encourage manufacturers to utilise the EAC-MRH procedure. Process of payment of fees by applicants should be addressed with the establishment of one central point for payment and decision making, which would make the process faster. Dedicated full-time staff should be appointed for the assessment of regional dossiers and the sustainability of the initiative will be enhanced if more technical officers are appointed
7) Regional Medicine Authority—The EAC Secretariat should re-consider the decision to establish a Regional Medicines Agency.

## 6 Conclusion

All agencies expressed the importance of the EAC-MRH work sharing initiative, especially with the current limited resources. The relevance of this initiative in the region cannot be over-emphasised, as it has enabled the regulatory institutions in the region with limited resources to continue to fight both sub-standard and falsified medical products and technologies. With the establishment of the African Medicines Agency, there is great hope that this continental authority will help shape the regional agencies. The EAC NRAs, African Union institutions, development partners and all stakeholders should be called on to mobilise resources that can improve the effectiveness and efficiency of the EAC joint assessment procedure. According to Ndomondo-Sigonda and colleagues, the problem of substandard and falsified medical products in Sub-Saharan Africa can only be addressed if the National Medicines Regulatory Authorities have the necessary support from their national governments and the public as well as a legal mandate to manage the regulation of medical products with the necessary human and financial resources (Ndomondo-Sigonda et al., 2020). To continuously improve this work-sharing and reliance initiative, the above key recommendations would need to be implemented at both national and regional levels.

## Data Availability

The dataset for this study is available upon request to the corresponding author.

## Abbreviations

ABREMA, Burundi Food and Medicines Regulatory Authority, Republic of Burundi; AMA, African Medicines Agency; AU, African Union; DFCA, Drug and Food Control Authority, Republic of South Sudan; EAC, East African Community; FDA, Rwanda Food and Drugs Authority, Republic of Rwanda; GMP, good manufacturing practice; MRH, Medicines Regulatory Harmonization; NDA, National Drug Authority, Uganda; NRAs, National Medical Regulatory Authorities; PPB, Pharmacy and Poisons Board, Republic of Kenya; RECs, regional economic communities; SAHPRA, South African Health Products Regulatory Authority; TMDA, Tanzania Medicines and Medical Devices Authority; WHO, World Health Organization; ZMDA, Zanzibar Medicines and Medical Devices Authority, the United Republic of Tanzania.

## References

[B1] Arik M. Bamenyekanye E. Fimbo A. Kabatende J. Kijo A. S. Simai B. (2020). Optimizing the East African Community's Medicines Regulatory Harmonization Initiative in 2020-2022: A Roadmap for the Future. Plos. Med. 17 (8), e1003129. 10.1371/journal.pmed.1003129 32785229 PMC7423061

[B2] CIRS (2021). R&D Briefing 82 – Regulatory Reliance Pathways: What Are the Opportunities and Barriers? London, UK: Centre for Innovation in Regulatory Science CIRS.

[B3] European Medicines Agency (2016). The European regulatory system for medicines. A consistent approach to medicines regulation across the European Union. London: European Medicines Agency. EMA/716925/2016.

[B4] Giaquinto A. R. Grignolo A. Liberti L. Lim J. C. W. Salmonson T. Sauer F. (2020). Improving Access to Quality Medicines in East Africa: An Independent Perspective on the East African Community Medicines Regulatory Harmonization Initiative. Plos. Med. 17 (8), e1003092. 10.1371/journal.pmed.1003092 32785224 PMC7423065

[B5] Keyter A. Salek S. Danks L. Nkambule P. Semete-Makokotlela B. Walker S. (2021). South African Regulatory Authority: The Impact of Reliance on the Review Process Leading to Improved Patient Access. Front. Pharmacol. 12, 699063. 10.3389/fphar.2021.699063 34366850 PMC8342884

[B6] Keyter A. Salek S. McAuslane N. Banoo S. Azatyan S. Walker S. (2020a). Implementation of a Framework for an Abridged Review Using Good Reliance Practices: Optimising the Medicine Regulatory Review Process in South Africa. Ther. Innov. Reg. Sci. 54, 1199–1207. 10.1007/s43441-020-00144-0 PMC7458939 32865802

[B7] Keyter A. Salek S. Banoo S. Walker S. (2020b). Can Standardisation of the Public Assessment Report Improve Benefit-Risk Communication? Front. Pharmacol. 11, 855. 10.3389/fphar.2020.00855 32625087 PMC7313675

[B8] Mashingia J. H. Ahonkhai V. Aineplan N. Ambali A. Angole A. Arik M. (2020). Eight Years of the East African Community Medicines Regulatory Harmonization Initiative: Implementation, Progress, and Lessons Learned. Plos Med. 17 (8), e1003134. 10.1371/journal.pmed.1003134 32785219 PMC7423058

[B9] Narsai K. Williams A. Mantel-Teeuwisse A. K. (2012). Impact of Regulatory Requirements on Medicine Registration in African Countries - Perceptions and Experiences of Pharmaceutical Companies in South Africa. South. Med. Rev. 5 (1), 31–37. 23093897 PMC3471191

[B10] Ncube B. M. Dube A. Ward K. (2021). Establishment of the African Medicines Agency: Progress, Challenges and Regulatory Readiness. J Pharm Policy Pract 14, 29. 10.1186/s40545-020-00281-9 33685518 PMC7938385

[B11] Ndomondo-Sigonda M. Mahlangu G. Agama-Anyetei M. Cooke E. (2020). A New Approach to an Old Problem: Overview of the East African Community's Medicines Regulatory Harmonization Initiative. Plos Med. 17 (8), e1003099. 10.1371/journal.pmed.1003099 32785223 PMC7423057

[B14] Roth L. Bempong D. Babigumira J. B. Banoo S. Cooke E. Jeffreys D. (2018). Expanding Global Access to Essential Medicines: Investment Priorities for Sustainably Strengthening Medical Product Regulatory Systems. Globaliz. Health 14 (1), 102. 10.1186/s12992-018-0421-2 PMC6211488 30382856

[B15] Sillo H. Ambali A. Azatyan S. Chamdimba C. Kaale E. Kabatende J. (2020). Coming Together to Improve Access to Medicines: The Genesis of the East African Community’s Medicines Regulatory Harmonization Initiative. PLoS. Med. 17 (8), e1003133. 10.1371/journal.pmed.1003133 32785273 PMC7423075

[B12] Sithole T. Mahlangu G. Capote V. Sitoie T. Shifotoka S. Gaeseb J. (2021a). Evaluation of the Review Models and Approval Timelines of Countries Participating in the Southern African Development Community: Alignment and Strategies for Moving Forward. Front. Med. 8, 742200. 10.3389/fmed.2021.742200 PMC8429484 34513894

[B13] Sithole T. Salek S. Mahlangu G. Walker S. (2021b). Comparison of the Registration Process of the Medicines Control Authority of Zimbabwe with Australia, Canada, Singapore, and Switzerland: Benchmarking Best Practices. Expert Rev. Clin. Pharmacol. 15, 109–119. 10.1080/17512433.2022.1987883 34645359

