# Supplementary material for: Evaluation of the Effectiveness and Efficiency of the East African Community Joint Assessment Procedure by Member Countries: The Way Forward
Source: Front Pharmacol. 2022 Jul 5;13:891506. doi: 10.3389/fphar.2022.891506 (PMC9294367; doi:10.3389/fphar.2022.891506)
Supplement: Supplementary file 1 [file DataSheet1.PDF]

Supplement 1: PROCESS EFFECTIVENESS AND EFFICIENCY RATING (PEER)

CONFIDENTIAL

EAC COLLABORATIVE MEDICINES REGISTRATION INITIATIVE PROCESS EFFECTIVENESS & EFFICIENCY RATING (PEER)

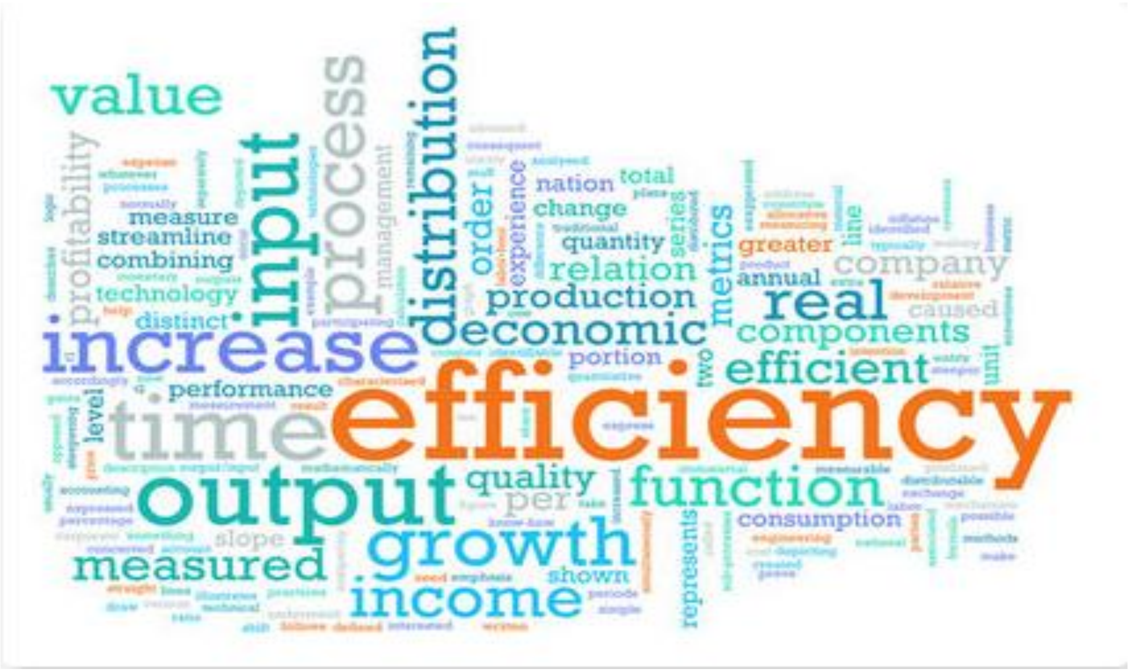

Pic taken from https://www.referenceforbusiness.com/management/De-Ele/Effectiveness-and-Efficiency.html

PEER QUESTIONNAIRE

August 2021

Contacts:

Nancy Ngum
nancyn@nepad.org

Prof Stuart Walker
swalker@cirs.org

Prof Sam Salek
sssalek52@gmail.com

INTRODUCTION

With the launch of the EAC-MRH project in 2012, countries have made substantial progress in reduction of timelines for registration of medical products using the joint review process. From 2012 to 2017 which has been considered as a pilot phase in a study by Mashingia et al,2020, registration timelines reduced from 24 months to 8 to 12 months for products reviewed using the new joint assessment process. Since 2017, there has not been a formal and structured evaluation of this work sharing programme and for its future direction, although some feedback has been sought through stakeholder meetings.

In recent years, there has been a drive within regulatory agencies to re-engineer their processes for improved efficiency and effectiveness and this often begins with a baseline evaluation of the current process to identify strengths and weaknesses. *Effectiveness* can be defined as 'doing the right thing' often measured by the value derived by customers/stakeholders from an organisation's processes or services while *Efficiency* can be defined as 'doing things right' which saves the organization time and resources.

Study Participants

The PEER Questionnaire is being sent to 6 National Medicines Regulatory Authorities of the EAC Member States namely, Pharmacy and Poisons Board, Republic of Kenya (PPB), National Drug Authority Uganda, Republic of Uganda (NDA), Rwanda Food and Drugs Authority, Republic of Rwanda (Rwanda FDA), Burundi (DPML), Drug and Food Control Authority, Republic of South Sudan (DFCA) and Zanzibar Food and Drug Authority, United Republic of Tanzania (ZFDA).

AIM

The aim of this study is to evaluate the effectiveness and efficiency of the current operating model of the EAC-MRH initiative including the challenges it faces as well as identifying opportunities for improvement.

STUDY OBJECTIVES

1. Obtaining the views of the individual medicines' regulatory authorities of the EAC-MRH initiative about the performance of the programme to date.
2. Identifying the challenges experienced by individual authorities throughout the life cycle of the EAC-MRH initiative.
3. Determining the strengths and weaknesses of the initiative
4. Identifying the ways of improving the performance of the work sharing programme.
5. Envisaging the strategy for moving forward

CONFIDENTIALITY

Thank you for agreeing to participate in this survey. **Your responses will be treated in strictest confidence and no identifiers of countries or respondents will be shared with any third party or made public**. External reports or presentations of the data will include only blinded results together with appropriate analytical interpretations.

The questionnaire is divided into five short sections and will take 20 minutes to complete. Thank you for taking time to complete it. We value your input!

A. DEMOGRAPHICS

1. Please state the name of your country _______
2. Please provide your responses to the following questions by writing your answer in the space provided or ticking the relevant box.
   a. Age: ____years
   b. Sex: ☐ Male ☐ Female
   c. Number of years of regulatory experience: _______years
3. What is the total number of staff in your agency? ___________
4. What is the number of reviewers of marketing authorization applications? _____________
5. How many reviewers participate in the EAC joint assessments? _______________
6. Does your agency have a separate record of applications received for assessment under EAC-MRH? ☐ Yes ☐ No

B. VIEWS ON THE BENEFITS OF THE EAC-MRH INITIATIVE
Select your answers by ticking the relevant box(es)

1. In your view, what are 3 (or more) benefits of the EAC-MRH initiative to date?

   ☐ Leadership commitment/Governance structure
   ☐ Clear Operating Model
   ☐ Shorter timelines for approval
   ☐ Information sharing among regulators
   ☐ Building of capacity for assessments
   ☐ Sustainable resource base because of self-funding by countries
   ☐ `Harmonisation of registration requirements across the region
   ☐ Other (Please specify) ____

2. What would you say are 3 (or more) strengths of your process for EAC-MRH products at country level that other countries could learn from?

   ☐ Separate register and tracking of EAC-MRH products
   ☐ Priority review of EAC-MRH products
   ☐ Information on the submission process and timelines for EAC-MRH products are available on your country website
   ☐ Products approved under EAC-MRH are available on your country website
   ☐ Regular Committee meetings enabling timely finalisation of products after EAC-MRH recommendation
   ☐ Other (Please specify) ____

3. How has the EAC-MRH initiative benefited member countries (regulators)?

   ☐ Training to improve the performance of the assessors
   ☐ Provides the platform for interaction and information exchange with other regulators
   ☐ Shared workload resulting in shorter timelines for approval than in individual countries
   ☐ Enables application of high standards of assessment regardless of size of country or maturity of regulatory agency
   ☐ Improved quality of dossiers submitted
   ☐ Other (Please specify) ____

4. How has the EAC-MRH initiative benefited manufacturers (applicants)?

- ☐ Reduced burden as they compile one dossier (modules 2 -5) for submission to multiple countries
- ☐ Savings on time and resources as they receive same list of questions from multiple countries enabling compilation of a single response package
- ☐ Shorter timelines for approval compared to that for the individual countries
- ☐ Access to various markets at the same time
- ☐ Other (Please specify) ____

5. How has the EAC-MRH initiative benefited patients in your country or in the EAC region?

- ☐ Quicker access to quality assured medicines
- ☐ Reduced prices of medicines
- ☐ Increased availability of medicines
- ☐ Other (Please specify) ____

C. VIEWS ON CHALLENGES OF THE EAC-MRH INITIATIVE

Select your answers by ticking the relevant box(es)

1. In your view, what are 3 (or more) challenges of the EAC-MRH initiative?

- ☐ Lack of detailed information on the process for applicants
- ☐ Low or decreasing number of applications for assessment
- ☐ Unequal workload among member countries
- ☐ Dependence on the countries' process for communication with applicants and expert Committees
- ☐ Lack of centralised submission and tracking
- ☐ Lack of jurisdiction power
- ☐ Other (please specify) ____

2. In your view, what are 3 (or more) challenges that you face at country level in assessing/finalising EAC-MRH products?

- ☐ Inadequate human resources
- ☐ Poor record keeping and tracking of EAC-MRH products
- ☐ Lack of priority review for EAC-MRH products
- ☐ EAC-MRH work not recognized as part of agency work to be done during working hours
- ☐ Unpredictable schedule of Committee meetings
- ☐ Lack of buy-in from expert Committee(s)
- ☐ Failure by manufacturers to follow the requirement to submit the exact same dossier to all countries of interest
- ☐ Failure by manufacturers to adhere to deadlines for response to questions
- ☐ Other (Please specify) ____

3. What are the challenges faced by manufacturers submitting applications to the EAC-MRH initiative?

- ☐ Differences in time to implementation of EAC-MRH recommendations by member countries.
- ☐ Lack of clarity about the process for submission and follow up in each country
- ☐ Lack of information on country websites and the EAC-MRH website about the process, milestones, timelines, pending and approved products
- ☐ EAC-MRH process is more stringent than some country processes
- ☐ Differing labeling requirements in participating countries
- ☐ Other (Please specify) ____

D. IMPROVING THE PERFORMANCE (EFFECTIVENESS AND EFFICIENCY) OF THE WORK-SHARING PROGRAMME

Select your answers by ticking the relevant box(es)

Effectiveness can be defined as 'doing the right thing' often measured by the value derived by customers/stakeholders from an organisation's processes or services while *Efficiency* can be defined as 'doing things right' which saves the organization time and resources.

1. What are 3 or more ways to improve the effectiveness of the EAC-MRH initiative in your view?

- ☐ Decision-making transparency e.g. publishing Public Assessment Reports
- ☐ Make publicly available any information that might help applicants in managing their submissions - templates of documents, lists of Q&A, timelines and milestones, disclosure of internal SOPs, etc.
- ☐ Consistency in application of guidelines and decisions
- ☐ Use of risk-based approaches e.g. reliance pathways
- ☐ Engagement and interaction with stakeholders
- ☐ Publishing of pending products
- ☐ Publishing of approved products
- ☐ Minimise the need for country specific documents
- ☐ Other (Please specify) ____

2. What are 3 or more ways to improve the efficiency of the EAC-MRH initiative in your view?

- ☐ Specific and clear requirements made easily available to applicants
- ☐ Compliance with target timelines by measuring and monitoring each milestone in the review process
- ☐ Use of robust IT systems
- ☐ Transparency on metrics and statistics e.g. % completed within timeline
- ☐ Improved central tracking of EAC-MRH products
- ☐ Improved resources e.g. number of assessors
- ☐ Centralised system for submission of applications and communication with applicants
- ☐ Other (please specify) ____

E: ENVISAGING THE STRATEGY FOR MOVING FORWARD

1. Rate the following proposals to improve the current EAC-MRH operating model from 1 – 3, number 1 representing what you think would be **most effective** in improving efficiency and number 3 the **least effective**. ***Enter the appropriate number in the space provided before each proposal.***

☐ To continue with the current operating model unchanged.

☐ To continue with the current operating model but provide full information on the process including timelines and milestones as well as approved products on every participating country's website and on the EAC-MRH website.

☐ The establishment of a regional administrative body to centrally receive and track EAC-MRH applications which would be responsible for allocating work, apportioning the applicable fees to countries, tracking of applications and communication with applicants.

2. In your view, would the establishment of an EAC regional medicines agency, if legally possible, be the best strategy for improved performance going forward? ☐ Yes ☐ No

   Please explain why?____

   ____

3. In conclusion, what other strategies not previously highlighted can you think of that would strengthen the EAC-MRH initiative going forward?

   ____

   ____

   ____

   ____

Please feel free to use the comment box below to elaborate on any of your answers or to highlight questions and answers that you believe should have been included in this questionnaire.

Name of person completing the questionnaire: ____________________________________

Title (position): _______________________________________

Date: __________________
Thank you for your time and help

SUPPLEMENT 2: INTERVIEW CHECKLIST - EAC PEER QUESTIONNAIRE

To determine the applicability, practicality, content validity and reliability of the responses in the questionnaire, the following questions were asked during the interview.

1. Are there any questions that you did not understand?
2. Is there any information you would like to add?
3. Were the questions relevant to the objectives of the survey?
4. In your opinion, what challenges did you encounter in completing the questionnaire?
5. Are there any other benefits and challenges of the EAC-MRH initiative that you think should be included in the questionnaire?
6. What is your general observation and remarks about this study?
7. What is its impact to the EAC Joint Assessment procedure?
